# Good Personality and Subjective Well-Being: Presence of Meaning in Life and Perceived Social Support as Mediators

**DOI:** 10.3390/ijerph192114028

**Published:** 2022-10-28

**Authors:** Xiaodan Xu, Yan Xu, Jinzhe Zhao, Panqin Ye, Mengke Yu, Yidie Lai, Juan Wang, Qunying Huang

**Affiliations:** 1 Beijing Key Laboratory of Applied Experimental Psychology, National Demonstration Center for Experimental Psychology Education (Beijing Normal University), Faculty of Psychology, Beijing Normal University, Beijing 100875, China; 2 Collaborative Innovation Center of Assessment toward Basic Education Quality, Beijing Normal University, Beijing 100875, China; 3 Vanke School of Public Health, Tsinghua University, Beijing 100084, China; 4 Student Mental Health Education Center, Neijiang Normal University, Neijiang 641100, China

**Keywords:** good personality, subjective well-being, presence of meaning in life, perceived social support

## Abstract

Background: Good personality is a positive moral personality in the context of Chinese Confucianism. Based on a social-cognitive model of normative well-being, we propose that good personality positively predicts subjective well-being, mediated by the perceived social support and presence of meaning in life in the context of Chinese culture. Methods: In this cross-sectional study, there were 665 Chinese adults (134 males and 531 females) who participated in the Good Personality Questionnaire, Multi-Dimensional Scale of Perceived Social Support, Presence of Meaning in Life Questionnaire, Positive and Negative Affect Scale, and Satisfaction with Life Scale. Results: Good personality was positively associated with subjective well-being (SWB). Both the presence of meaning in life and perceived social support independently mediated the link between good personality and subjective well-being (SWB), and in Chinese adults, perceived social support has a greater mediating effect than the presence of meaning in life. Conclusion: These findings illustrate that the presence of meaning in life and perceived social support mediate the relationship between good personality and subjective well-being in the context of Chinese culture, which supports the model of normative well-being and can provide more targeted intervention guidance for research on promoting well-being in the Chinese context.

## 1. Introduction

Subjective well-being (SWB) can be generally defined as optimal psychological functioning [1,2]. It is a complex construct that is crucial for both mental and physical wellness [3,4]. SWB consists of three separate components: life satisfaction, positive emotions, and negative emotions. Generally speaking, life satisfaction is the cognitive component of subjective well-being, which refers to an individual’s subjective assessment of their life. Positive emotions minus negative emotions measure the emotional component of well-being [4]. Many studies have found that positive personality traits contribute to SWB [5,6,7,8,9]. However, many contemporary personality theories are based on Western cultural, philosophical, and sociological assumptions about people [10]. In turn, this makes it difficult for psychologists to understand the cultural differences in personality models. With Western theories, concepts, and tools, psychologists have attempted to compare differences between individuals living in different parts of the world. In this kind of approach, people are judged based on culturally incompatible standards. In contrast, according to a cultural-psychology approach, personality is entirely influenced by the meanings and practices of a particular sociocultural context [10]. Therefore, a Chinese personality concept is used in this paper in order to explain the differences between Chinese people in terms of their daily lives. In addition, moral character is a positive trait with a specific moral component, but there has been little research on its impact on SWB. The moral character of humans may differ from that of other species in a unique way [11,12], and morality is at the heart of "important life events, responses to the environment, and the impact on well-being" [13]. Therefore, there is a need to examine the impact of positive moral character on SWB. Accordingly, we focus on the influence of Confucianism’s positive moral personality trait of-good personality-on subjective well-being of the Chinese.

The concept of good personality is unfamiliar to English speakers. The significance of its role in Chinese culture must be demonstrated. The Confucian philosophy is a key to understanding China [14]. The classics of Confucianism likely have a greater influence on Chinese people than any other literary or philosophical works. In the long history of Chinese culture, Confucianism has subtly influenced generations of people. The Confucian philosophers, represented by Confucius (551-479 BCE) and Mencius (372-289 BCE), held goodness in high esteem, proclaiming the idea of “benevolence” and advocating “benevolent government”. The opinions of Confucius and Mencius on the inherent goodness of human nature and of the Confucian philosopher Seosso (date of birth and death unknown) on the intrinsic good and evil of human nature have laid the core cultural and psychological foundation of “benevolence” in China. This cultural essence of goodness is also engraved in the collective memory of the Chinese people, influencing their nationality and national character and creating the basic structure of their personality [15]. The good personality is a theme of personality psychology research with Chinese cultural characteristics. Therefore, good personality is an excellent gateway to understanding Chinese individual differences.

According to the social-cognitive model of normative well-being, personality, cognitive, and environmental support have critical contributions to well-being. It emphasizes that cognitive and environmental variables may mediate personality’s effects on well-being [16]. Additionally, based on empirical evidence, the model explains how social and cognitive variables work together to maintain well-being in normal circumstances [17,18]. In the Chinese cultural context, people value their connections with the outside world and expect to be valued for their social relationships and support [19,20,21]. Therefore, the variable of environmental support in this study was chosen to perceived social support. Meaning in life is a fundamental need for human existence. People need to construct meaning for their own rules of reference in order to combat the anxiety that life will eventually end. The cognitive dimension of the meaning of life provides a holistic structure for people to understand themselves, the world, and their relationship with it [22]. Further, the presence of meaning in life (“having meaning”) is the extent to which individuals feel that they are living a meaningful life based on their understanding of the meaning and purpose of life. This is commonly referred to as the “sense of meaning” or “experience of meaning”. It has a cognitive dimension with an emphasis on outcomes [23]. Therefore, this study chose the presence of meaning in life as a cognitive variable. In this study, the effect of a positive moral character-good personality-on SWB is examined by investigating whether good personality in the Chinese cultural context contributes to the cognitive and social resources associated with the presence of meaning in life and perceived social support, and subsequently with subjective well-being.

### 1.1. Good Personality and Subjective Well-Being (SWB)

Hillson (1999) [24] believes that personality traits are divided into positive and negative. Based on an evolutionary perspective, some scholars have suggested that humans can be both good and evil, altruistic and selfish. Only a combination of both good and evil traits can drive evolution [25]. Previous research has also found that individuals have personality sub-types with light and dark characteristics [26]. This means that the individual may have both a dark and a light personality [27]. Previous research has shown that positive personality traits such as high extraversion, high openness, and high commitment increase the use of problem-focused coping strategies (e.g., positive reappraisal strategies) when individuals are faced with stress [28]. Positive personality traits can have a beneficial effect on mental health and well-being [29,30]. In times of stress, people with negative personality traits are more likely to use emotion-centered coping strategies [31,32]. Previous studies on the relationship between negative personality and well-being have reached inconsistent conclusions. For example, narcissism was positively associated with subjective well-being [33], psychopathy was negatively related to subjective well-being [33], Machiavellian personality was negatively associated with subjective well-being [33], or was unrelated to well-being [34].

There is a great deal of concern about a person’s moral character [35], which comprises the moral dimensions of their personality [36]. It has been found that moral personality in the Chinese cultural context was also divided into positive and negative. Positive moral character (good personality) refers to the nature of a person that determines whether they will be helpful to others; negative moral character (evil personality) refers to the nature of a person that determines whether they will be harmful to others [37,38]. It means each person may have a mixture of positive (good personality) and negative (evil personality) moral personality traits and the mutually independent structures of good and evil personality [37,38]. Studies have found consistent and stable results for positive personality in promoting subjective well-being [25,26,27,28,29]. Therefore, this study focuses on the role and mechanisms of positive moral personality-good personality-in subjective well-being.

The good personality has the core characteristics of structure, disposition, sociality, and morality [37,38]. Structure refers to the sense that the good personality has four dimensions: conscientiousness, altruism, amicability, and magnanimity [37,38]. Disposition refers to the fact that individuals of different good personality have a specific moral bias in expressing their words and actions [37,38]. According to the dual processing system theory, people have two systems of deliberate processing and intuitive processing to respond. Deliberate processing (controlled processing) is a slow, mental resource-consuming, while intuitive processing is a fast, low-energy-consuming, heuristic processing that individuals perform according to their true inner thoughts and tendency to respond [39]. That is, good people commit acts of kindness as a spontaneous, intuitive, and favorable response. On the whole, although good personality does not entirely determine the behavior of individuals, it is an intrinsic psychological quality with social and moral evaluation significance. It can, to a certain extent, reflect the different tendencies of individuals in cognitive processing and behavioral performance. In addition to its structural and dispositional characteristics, good personality also has the core characteristic of sociality, which means that the formation of the good personality of an individual is not only related to the innate biological basis but also has a great relationship with the living environment, cultural atmosphere, etc. [34,35,36]. The formation of a good personality is influenced by a variety of social factors, while at the same time, personality plays a role in social perceptions and responses. “Morality” means that the actions described, explained, and predicted by the good personality have moral characteristics and can be evaluated as morally good or bad, reflecting mainly the critical aspects of the good personality [37,38]. People usually access good and evil based on the morality of the behavior and the tendencies behind it.

As mentioned before, a few studies have explored the effects of positive moral character on well-being. Based on the developmental assets framework and Lerner’s 5C/6C model [40], positive moral character shapes SWB [41]. Only one study in the Chinese cultural context found that positive moral character positively predicts adolescents’ life satisfaction [42]. It is unclear whether moral character influences adults’ well-being. Therefore, this paper explores whether good personality in the Chinese cultural context promotes adults’ subjective well-being, which is very important in enhancing the happiness of Chinese people.

The dimensions of good personality (e.g., conscientiousness and altruism) are related to SWB. It has been suggested that conscientiousness contributes to well-being [5]. Additionally, the results of previous studies indicate a dual-path model in which altruism increases adaptability. The internal path refers to the fact that altruism can promote the altruist’s positive physical and mental interaction through the internal process of self-incentive and bring internal utility gain, which improves the adaptability of the altruist at some moments. The external pathway is helpful in enhancing group status, cooperation, and mate selection. Thus, altruism can be preserved by evolutionary selection at the individual and group levels [43,44]. Thus, it is worth pursuing an examination of the effects of good personality on subjective well-being, and we have formulated the following hypothesis:

**Hypothesis** **1** **(H1).** *A positive relationship exists between good personality and subjective well-being*.

### 1.2. Good Personality, Perceived Social Support, and Subjective Well-Being

Perceived social support is the individual’s view of the availability and satisfaction of provided support [45]. Considerable evidence has shown that a positive personality is positively associated with perceived social support [46,47], and the core qualities of moral character are considered essential to social relations [48]. In other words, individuals with good personality intended to be more helpful to others, which contributes to positive social relations or more social support. In addition, the dimension of good personality (e.g., conscientiousness) is positively related to perceived social support [49,50]. Therefore, we would like to suppose that good personality should be positively associated with perceived social support.

In addition, Chinese culture is a collectivist one. People in collectivist societies place greater emphasis on social harmony [18] and prefer to connect with others and relate their interests to the interests of others [19,20]. As a result of relationships, adults perceive value and support, which is a critical component of SWB [51]. Furthermore, the convoy model of social relations suggests that perceived social support is vital in individual SWB [52]. Previous researches have identified three main theories of the role of social support in mental health: the main effect model, buffering model, and the dynamic model. The main effect model holds that social support has a universal beneficial effect on an individual’s physical and mental health. It is not limited to stressful circumstances. It is to maintain the individual’s good emotional experience and physical and psychological condition at ordinary times to be beneficial to physical and mental health. There is a direct correlation between the level of social support and the level of physical and mental health. The buffering model holds that social support plays a role in individuals’ physical and mental health through the elimination of stress. Individuals’ physical and mental health can be maintained and improved by buffering the adverse effects of stressful events. A dynamic effect model assumes that stress and social support affect physical and mental health directly or indirectly. There is a mutual influence and interaction between social support and stress, and this relationship will change over time [53]. Whichever model is used, it is clear that social support is essential for physical and mental health. Furthermore, people with higher perceived social support tend to have a stronger sense of SWB, according to an empirical study [54]. Moreover, it has been demonstrated that perceived social support mediates the relationship between positive traits and SWB [46,55]. In light of this, we propose the following hypotheses:

**Hypothesis** **2** **(H2).** *Good personality and perceived social support have a positive relationship*.

**Hypothesis** **3** **(H3).** *Perceived social support and subjective well-being form a positive relationship*.

**Hypothesis** **4** **(H4).** *Perceived social support mediates the relationship between good personality and subjective well-being*.

### 1.3. Good Personality, Presence of Meaning in Life, and Subjective Well-Being (SWB)

In terms of the presence of meaning, it refers to how much people perceive their lives to be meaningful and how much they value their lives for higher purposes than their mundane ones [56]. It has been found that positive personalities (e.g., extraversion and agreeableness) possess a positive association with the presence of meaning [11,57], and forgiveness can promote meaning in life [58]. In particular, the subdimensions of good personality (conscientiousness and altruism) are both positively correlated with the presence of meaning [59,60]. Therefore, the relationship between good personality and the presence of meaning is supposed to be positive.

The theory of meaning maintenance defines many cognitive skills and dimensions that contribute directly to our sense of well-being by maintaining our meaning, feelings, and life’s purposes [22]. Research findings have generally shown that the presence of meaning is associated with increased levels of SWB [61,62]. This means that a higher level of presence of meaning has been shown to improve well-being [63]. A meta-analytic study also found a strong link between the presence of meaning and SWB [46]. It has also been believed that the presence of meaning can indirectly contribute to SWB by enhancing correlates that are important to SWB [64]. Either way, previous studies have largely concluded that the presence of meaning is a robust factor correlated with SWB. Therefore, the literature clearly shows a link between positive personality, presence of meaning, and SWB. As a valuable personal resource, presence of meaning may be able to mediate the relationship between good personality and SWB. Therefore, we proposed the following hypotheses:

**Hypothesis** **5** **(H5).** *A positive relationship exists between good personality and presence of meaning in life*.

**Hypothesis** **6** **(H6).** *A positive relationship exists between the presence of meaning in life and subjective well-being*.

**Hypothesis** **7** **(H7).** *Presence of meaning in life mediates the relationship between good personality and subjective well-being*.

### 1.4. Current Study

Despite a large body of available research, we know little about the role and mechanism of good personality in explaining SWB in the context of Chinese culture. Therefore, we investigated how good personality affects SWB using a normative well-being model and how perceived social support and presence of meaning mediate it.

The present study attempted to address the following problems. First, this study aimed to explore the effect of good personality on SWB in the context of Chinese culture. Second, this study aimed to investigate the mediating roles of presence of meaning and perceived social support on good personality and SWB. Based on the normative well-being model, we developed a research framework to examine how presence of meaning and perceived social support explain the link between good personality and SWB.

## 2. Materials and Methods

### 2.1. The Participants and the Procedure

In total, 665 Chinese adults (average age = 21.97 years, SD = 4.68) were involved in this research, ranging in age from 18 to 57 years. Fourteen participants did not report their age (2.10%), 317 were between 18 and 20 years (47.67%), 242 between 21 and 25 years (36.24%), and 106 over 25 years (15.94%). In terms of gender, the respondents included 134 males (20.15%) and 531 (79.85%) females; subjective socioeconomic status ranged from 1 to 10 (M = 6.10, SD = 1.52). Handy sampling was used in this study. We shared an online research link through Moments of WeChat. This study was conducted through questionnaires distributed online. Participants were informed that their responses would remain confidential so that they could complete the survey honestly. After finishing the informed consent, the participants completed a multi-section questionnaire online, which included the measures of good personality, perceived social support, presence of meaning, and SWB. An ethics committee approval was obtained for this study at Beijing Normal University.

### 2.2. Psychological Measures

#### 2.2.1. Good Personality Questionnaire

The Good Personality Questionnaire measured the participants’ good personality [34]. It consisted of 15 items and measured four components of Chinese good personality: conscientiousness, altruism, amicability, and magnanimity. The participants answered on a Likert scale from 1–5; 1 indicated strongly disagree, and 5 indicated strongly agree. The Good Personality Questionnaire was reliable and valid [37]. Cronbach’s alpha coefficient of 0.96 and Cronbach’s Omega of 0.90 were obtained; conscientiousness, altruism, amicability, and magnanimity had Cronbach’s alpha coefficients of 0.94, 0.87, 0.89, and 0.86, and Cronbach’s Omega of 0.84, 0.86, 0.89, and 0.84, respectively.

#### 2.2.2. Satisfaction with Life Scale (SWLS)

The SWLS measured the participants’ overall life satisfaction [4]. There were five items, and a scale of 1-7 was used to measure global life satisfaction, with 1 indicating very strongly disagree, and 7 indicating very strongly agree. This scale has repeatedly been found in China to have good psychometric properties among populations [65]. Based on our data, the Cronbach’s alpha coefficient of SWLS was 0.84, and the Cronbach’s Omega coefficient was 0.89.

#### 2.2.3. Positive and Negative Affects Scale (PANAS)

Nine positive and nine negative affections from the PANAS were used to measure the participants’ positive and negative affects [66]. Each item was rated on the frequency over the past month on a 1–5 Likert scale. 1 was not at all, and 5 was extremely. Positive and negative affects had Cronbach’s alpha coefficients of 0.95 and 0.89 and Cronbach’s Omega coefficients of 0.96 and 0.91, respectively.

#### 2.2.4. Presence of Meaning in Life Questionnaire

The participants completed answers to the Presence of Meaning in Life Questionnaire [56]. There were five items rated on a 1–7 Likert scale, with 1 indicating very strong disagreement, and 7 indicating very strong agreement. Studies have demonstrated the good psychometric properties of the scale when used with Chinese colleges [26], and according to our data, a Cronbach’s alpha coefficient of 0.86 and a Cronbach’s Omega coefficient of 0.90 were obtained.

#### 2.2.5. Multidimensional Scale of Perceived Social Support (MSPSS)

The MSPSS [67] was used to evaluate perceived social support. The MSPSS included 12 items to measure friend, family, and other support. Likert scales were used to score each item, with 1 indicating very strong disagreement and 7 indicating very strong agreement. We found that the Cronbach’s alpha coefficient and Cronbach’s Omega coefficient were 0.92 and 0.93, respectively in our study, and the Cronbach’s alpha coefficients of friend support, family support, and other support were in the following order: 0.88, 0.90, and 0.88, while their Cronbach’s Omega coefficients were in the following order: 0.92, 0.93, and 0.92.

### 2.3. Statistical Analysis

IBM SPSS 25 and Mplus7 were used to analyze the data. First, we analyzed the correlations between the main variables with IBM SPSS 25. Then, we examined the model fit to determine the relationships among SWB, good personality, presence of meaning, and perceived social support using structural equation modeling (SEM). Presence of meaning is a unidimensional structure and is constructed by using its original items as indicators. The remaining variables are multidimensional and use an item parceling strategy; they were built by using all of their dimensions as indicators. In addition to the root mean square error of approximation (RMSEA) and standardized root mean squared residual (SRMR), the Tucker–Lewis index (TLI) and comparative fit indices (CFI) were used to evaluate the fit of the model. The fit of a model can be assessed by RMSEA and SRMR values below 0.08 and CFI values above 0.9 [68,69]. The nonparametric bootstrap method tested the mediation effects, and a 95% CI containing no zero was considered significant [70].

## 3. Results

### 3.1. Confirmatory Factor Analysis

Using IBM SPSS 25 and Mplus7, we first performed series-wise confirmatory factor analysis to examine the difference between the scales in terms of good personality, perceived social support, presence of meaning in life, and subjective well-being.

There is a better fit to the data with the proposed four-factor model than any alternative, as seen in Table 1, which presents RMSEA = 0.05; SRMR = 0.04; CFI = 0.96; TLI = 0.94; *χ*^2^/*df* = 2.79, *p* < 0.001. Analyses show that this research is best suited for a four-factor model.

Four latent variables (good personality, presence of meaning, perceived social support, and SWB) and 16 observed variables formed the measurement model. A significant factor loading was observed for each indicator (*ps* < 0.001), which indicated that the observed indicators adequately reflected the four latent variables, as seen in Table 2. The measurement model satisfactorily fit the data: *χ*^2^ = 237.87, *df* = 84, *p* < 0.001; RMSEA = 0.05; SRMR = 0.04; CFI = 0.96; TLI = 0.95.

The AVE and CR values were also calculated in this research, and the results showed that the AVE value of subjective well-being was barely within the acceptable range due to the low loading coefficient of negative emotions. However, since SWB is a classic three-factor model [4], this paper used three components to calculate SWB in reference to previous studies [6,49].

### 3.2. Analyses of Descriptive Statistics and Correlations

The means, standard deviations, and correlation coefficients for the variables are listed in Table 3. Good personality was positively associated with perceived social support (r = 0.40, *p* < 0.001), presence of meaning (r = 0.35, *p* < 0.001), and SWB (r = 0.43, *p* < 0.001). Perceived social support was positively associated with presence of meaning (r = 0.44, *p* < 0.001) and SWB (r = 0.62, *p* < 0.001). Presence of meaning was positively associated with SWB (r = 0.48, *p* < 0.001).

### 3.3. Multiple Mediation Results

Structural Model 1 was established with good personality taking into account the independent variable, subjective well-being taking into account the dependent variable, and perceived social support and presence of meaning as mediators (see Table 4 and Table 5). Model 1 fitted well to the data (*χ*^2^ = 330.02, *df* = 85, *p* < 0.001; RMSEA = 0.07; SRMR = 0.07; CFI = 0.94; TLI = 0.93).

In Table 4, good personality was found to have a significant effect (β = 0.15, *p* < 0.05) on subjective well-being. Hypothesis 1 was supported. Hypotheses 2 and 5 were also found to be supported. Furthermore, perceived social support had a significant effect (β = 0.57, *p* < 0.001) on subjective well-being, as did the presence of meaning in one’s life (β = 0.36, *p* < 0.001).

Table 5 shows the results using the bootstrap bias-correction approach with 5000 samples taken to investigate Hypotheses 4 and 7 that perceived social support (95%CI = [0.23, 0.39]) and presence of meaning (95% CI = [0.12, 0.25]) independently mediated the link between good personality and subjective well-being.

**Figure 1:**
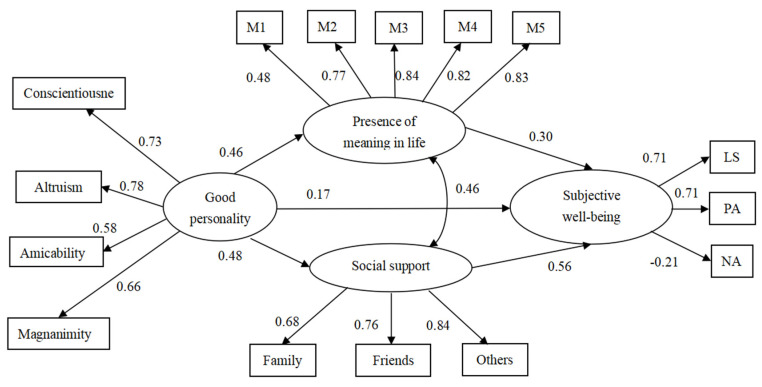
Finalized structural model (N = 665). Factor loading is standard. M1–M5 = five items of presence of meaning in life; PA = positive affect; NA = negative affect; LS = life satisfaction.

Because presence of meaning has been shown to be positively associated with social support [71], presence of meaning was allowed to be linked to perceived social support in model 2, and the model fitted the data well (*χ*^2^ = 237.87, *df* = 84, *p* < 0.001; RMSEA = 0.05; SRMR = 0.04; CFI = 0.96; TLI = 0.95). Model 2 had better performance than Model 1 (*χ*^2^ = 92.15, *df* = 1, *p* < 0.05). Therefore, the finalized structural model was completed based on model 2 (Figure 1).

As shown in Table 6, Hypothesis 1–3 and Hypotheses 5 and 6 were also supported in model 2. Table 7 shows that both perceived social support (95% CI = [0.19, 0.34]) and presence of meaning (95% CI = [0.08, 0.19]) had independent mediated effects on the link between good personality and SWB. Additionally, we tested an effect contrast, and perceived social support had a more substantial mediation effect than presence of meaning (95% CI = [0.05, 0.39]).

## 4. Discussion

We examined the relationship between good personality and SWB and the roles of both presence of meaning and perceived social support as mediators. As hypothesized, the findings found that good personality was positively associated with SWB and that the link was mediated independently by presence of meaning and perceived social support, which was consistent with Lent’s normative model of well-being [54]. Moreover, presence of meaning and perceived social support had different mediation effects, and perceived social support had a greater mediating effect than presence of meaning. Accordingly, the results suggest that presence of meaning and perceived social support serve as critical mediators of how good personality influences SWB.

First, in our study, there was a positive correlation between good personality and SWB among 665 Chinese adults, which is in accordance with previous research [40,41,42,72]. Using the Model of Developmental Assets, good personality in Chinese culture falls into the category of “positive values” contributing to SWB in adulthood [40]. In general, good personality in the context of Chinese culture positively predicts SWB, as do positive personalities, and moral character is a positive predictor of SWB. Although there are more previous studies on positive personality for well-being [40,41,42,72], there are fewer studies on positive moral personality for subjective well-being [41], especially those specific to the Chinese cultural context [42]. This study not only enriches the field of research in the Chinese cultural context but also serves as a slight reference for subsequent research in this area. In addition, it can provide more targeted intervention guidance for research on promoting well-being in the Chinese context. The positive or moral personality that has previously promoted subjective well-being has originated chiefly in Western countries. However, good personality is a positive moral personality rooted in Chinese culture, and interventions to promote subjective well-being through developing of good personality are more targeted and potentially more effective.

Second, perceived social support was found to be one of the mediators between good personality and SWB. As hypothesized, good personality is positively related to perceived social support. This study confirms that positive traits are linked to SWB through perceived social support [46]. When people perceive and evaluate others, the moral character may be a predominant dimension [36] because people want to know whether others are inclined to have the necessary qualities that they expect in a social relationship and whether to plan for social investment or not [36,48]. Therefore, having a good personality leads to adults accepting more social investments, forming positive relationships, and reporting higher perceived social support scores, which may contribute to higher SWB levels.

Third, we found a mediating effect of presence of meaning on the good personality and SWB relationship. A person’s character that determines whether they will be supportive of others is referred to as good personality [37]; individuals with a higher good personality level are more likely to help others (e.g., prosocial behavior; altruistic behavior). Consequently, they may have a stronger sense of meaning in life [73]. Furthermore, many cognitive skills are required for one to feel meaningful and purposeful and achieve meaning in life. Adults with high levels of good personality may build more inner cognitive resources-presence of meaning-that assist them in achieving high levels of SWB [22]. Therefore, adults with good personality could promote their SWB through presence of meaning.

The presence of meaning and perceived social support showed significant differences in the mediation effects of good personality on SWB; in Chinese adults, perceived social support has a more significant mediating effect than presence of meaning. Perhaps this is due to China’s being a collectivist cultural state; compared with individualistic cultures, people in collectivist countries emphasize social harmony and consider relatedness more highly [18]. Collectivists prefer to connect with others and relate their interests to those of groups [19]. Chinese people need personal relationships because of collectivist values [20]. Relationships lead adults to perceive value and support, which is a core factor of SWB [51].

Our research has made several notable contributions. In theoretical terms, this study provides oriental insights into subjective well-being. Confucianism stresses the importance of goodness to happiness, believing that good deeds lead to happiness and that “goodness is the foundation of happiness”. If one does not possess goodness, one cannot attain happiness. The process of constantly improving one’s goodness is the pursuit of happiness, and the highest happiness is achieved when goodness is perfected. The second important feature of this study is the use of culturally compatible personality concepts and theoretical frameworks stemming from classical philosophy to account for individual differences. These findings support Jiao’s studies [37,38], indicating good personality is a valid construct. Third, the normative well-being model is validated in the Chinese cultural context. Consistent with the model, good personality helps individuals to develop internal cognitive resources and environmental resources, which promote a sense of well-being. It also demonstrates the importance attached to relationships in a collectivist culture. In practical terms, this study can guide more targeted interventions for people’s well-being in China, such as development programs that can build cognition sources (e.g., presence of meaning in life) and environmental sources (e.g., social support from family, friends, strangers, etc.). In addition, the results may have a certain guiding effect on inducing people to be good. Being a good person may lead to a higher sense of life meaning and social support, which contribute to a higher sense of well-being, and this may make people more willing to be good people. The results of this study have certain reference significance for moral education and personality education.

There are several potential limitations to this study. Although the questionnaires chosen for this study have good psychometric characteristics, they all rely on self-reporting. Attempts were also made to control for response biases by using some self-reported measures as covariates, but further studies are needed to rule out the biases caused by other factors. Second, most of the study samples were collected from college students, and the stability of the results in different age groups needs to be further verified. Third, it was impossible to identify a causal relationship between the variables in this study. In the future, longitudinal or laboratory studies will be necessary to determine the causality between the variables.

## 5. Conclusions

According to our findings, Chinese adults showed an association between good personality and SWB through the presence of meaning and perceived social support, which the normative well-being model may explain. Besides enriching research on moral character, the results validate the Social Cognitive Model of SWB in a Chinese cultural context. In addition, our findings have crucial clinical implications by providing valuable guidance for developing interventions related to good personality to promote subjective well-being and for developing projects that, for instance, can build presences of meaning in life and perceived social support.

## Figures and Tables

**Table 1:** Confirmation factory analysis.

Model	*χ* ^2^	*df*	*χ*^2^/*df*	RMSEA	SRMR	CFI	TLI
Four-factor model ^a^	239.49	86	2.79	0.05	0.04	0.96	0.94
Three-factor model ^b^	739.03	87	8.50	0.11	0.10	0.84	0.82
Two-factor model ^c^	981.24	89	11.03	0.12	0.13	0.78	0.77
One-factor model ^d^	1289.45	90	13.99	0.14	0.11	0.71	0.70

N = 665. ^a^ Good personality, perceived social support, presence of meaning in life, and subjective well-being combined together as one construct. ^b^ Two mediators (perceived social support and presence of meaning in life) combined as one construct; good personality and subjective well-being combined as one construct. ^c^ Two mediators (perceived social support and presence of meaning in life) combined as one construct, with good personality and subjective well-being as separate constructs. ^d^ Good personality, perceived social support, presence of meaning in life, and subjective well-being as separate constructs.

**Table 2:** Confirmatory factor analysis results for the four-factor model.

Latent Variable		Estimate	C.R.	Standardized Estimate (λ)	R-Square	AVE	Composite Reliability
Good personality	Conscientiousness	1	-	0.73 ***	0.53	0.48	0.78
	Altruism	1.15	16.50	0.78 ***	0.61		
	Amiability	0.96	13.18	0.58 ***	0.34		
	Magnanimity	1.28	14.82	0.66 ***	0.44		
Presence of meaning in life	M1	1	-	0.48 ***	0.23	0.58	0.87
	M2	1.37	12.17	0.77 ***	0.59		
	M3	1.47	12.60	0.84 ***	0.71		
	M4	1.38	12.51	0.82 ***	0.68		
	M5	1.57	12.55	0.83 ***	0.69		
Perceived social support	Family	1	-	0.68 ***	0.47	0.59	0.81
	Friends	0.93	16.64	0.76 ***	0.58		
	Others	1.19	17.64	0.84 ***	0.71		
Subjective well-being	LS	1	-	0.71 ***	0.50	0.35	0.43
	PA	0.87	15.26	0.71 ***	0.50		
	NA	−0.21	−4.88	−0.21 ***	0.05		

Note: C.R.: critical ratio, AVE: average variance extracted. *** *p* < 0.001.

**Table 3:** Means, SD, and correlations for the main study variables (N = 665).

	*M*	*SD*	1	2	3	4
1. good personality	3.50	0.55	1			
2. perceived social support	4.83	1.04	0.40 ***	1		
3. presence of meaning	4.74	0.88	0.35 ***	0.44 ***	1	
4. subjective well-being	5.10	1.94	0.43 ***	0.62 ***	0.48 ***	1

Note: *** *p* < 0.001.

**Table 4:** SEM Results for Model 1.

Hypotheses		Estimate	S.E.	β
H1	Good personality → Subjective well-being	0.25	0.11	0.15 *
H2	Good personality → Perceived social support	1.08	0.13	0.54 ***
H3	Perceived social support → Subjective well-being	0.50	0.05	0.57 ***
H5	Good personality → Presence of meaning in life	0.91	0.13	0.51 ***
H6	Presence of meaning in life → Subjective well-being	0.33	0.06	0.36 ***

Note: S.E.: Standard error, β: Standardized coefficients. * *p* < 0.05; *** *p* < 0.001.

**Table 5:** Results of indirect effect analysis of Model 1.

Relationship of Variables		Indirect Effect	Lower	Upper
	Indirect Effect			
H4	GP → PSS → SWB	0.31 ***	0.23	0.39
H7	GP → POM → SWB	0.19 ***	0.12	0.25
	Total Indirect Effect	0.50 ***	0.39	0.56
	Direct Effect	0.15 **	0.03	0.28
	Total Effect	0.65 ***	0.55	0.75

GP: Good personality, POM: Presence of meaning in life, PSS: Perceived social support; SWB: Subjective well-being. ** *p <* 0.01, *** *p <* 0.001.

**Table 6:** SEM Results for Model 2.

Hypotheses		Estimate	S.E.	β
H1	Good personality → Subjective well-being	0.29	0.10	0.17 **
H2	Good personality → Perceived social support	0.97	0.12	0.48 ***
H3	Perceived social support → Subjective well-being	0.46	0.05	0.56 ***
H5	Good personality → Presence of meaning in life	0.82	0.12	0.46 ***
H6	Presence of meaning in life → Subjective well-being	0.28	0.06	0.30 ***

Note: S.E.: Standard error, β: Standardized coefficients. ** *p* < 0.01, *** *p* < 0.001.

**Table 7:** Results of indirect effect analysis of Model 2.

Relationship of Variables		Indirect Effect	Lower	Upper
	Indirect Effect			
H4	GP → PSS → SWB	0.27 ***	0.19	0.34
H7	GP → POM → SWB	0.14 ***	0.08	0.19
	Total Indirect Effect	0.41 ***	0.32	0.49
	Direct Effect	0.17 **	0.06	0.29
	Total Effect	0.58 ***	0.48	0.68

GP: Good personality, POM: Presence of meaning in life, PSS: Perceived social support; SWB: Subjective well-being. ** *p* < 0.01, *** *p* < 0.001.

## Data Availability

Not applicable.

## References

[1] King M.L. (2019). The neural correlates of well-being: A systematic review of the human neuroimaging and neuropsychological literature. Cogn. Affect. Behav. Neurosci..

[2] Lyubomirsky S., Sheldon K.M., Schkade D. (2005). Pursuing Happiness: The Architecture of Sustainable Change. Rev. Gen. Psychol..

[3] Van Lente E., Barry M.M., Molcho M. (2012). Measuring population mental health and social well-being. Int. J. Public Health..

[4] Diener E., Emmons R., Larsen R., Griffin S. (1985). The Satisfaction with Life Scale. J. Personal. Assess..

[5] Mueller S., Wagner J., Wagner G.G., Ram N., Gerstorf D. (2019). How far reaches the power of personality? Personality predictors of terminal decline in well-being. J. Personal. Soc. Psychol..

[6] Anglim J., Horwood S., Smillie L.D., Marrero R.J., Wood J.K. (2020). Predicting psychological and subjective well-being from personality: A meta-analysis. Psychol Bull..

[7] Aghababaei N., Błachnio A., Arji A., Chiniforoushan M., Tekke M., Mehrabadi A.F. (2016). Honesty–humility and the HEXACO structure of religiosity and well-being. Curr. Psychol..

[8] Aghababaei N., Arji A. (2015). Well-being and the HEXACO model of personality. Personal. Individ. Differ..

[9] Aghababaei N. (2014). God, the good life, and HEXACO: The relations among religion, subjective well-being and personality. Mental Health. Relig. Cult..

[10] Markus H.R., Kitayama S. (1998). The cultural psychology of personality. J. Cross-Cult. Psychol..

[11] Haslam N. (2006). Dehumanization: An Integrative Review. Personal. Soc. Psychol. Rev..

[12] Haslam N., Bain P., Douge L., Lee M., Bastian B. (2005). More human than you: Attributing humanness to self and others. J. Personal. Soc. Psychol..

[13] Prentice M., Jayawickreme E., Hawkins A., Hartley A., Furr R.M., Fleeson W. (2019). Morality as a basic psychological need. Soc. Psychol. Personal. Sci..

[14] Allan S. (2000). Introduction. The Analects..

[15] Li H., Chen A.T. (2003). On the Chinese Basic Personality Structure Influenced by Benevolence. J. Southwest China Norm. Univ. (Humanit. Soc. Sci. Ed.).

[16] Lent R.W. (2004). Toward a Unifying Theoretical and Practical Perspective on Well-Being and Psychosocial Adjustment. J. Couns. Psychol..

[17] Garriott P.O., Hudyma A., Keene C., Santiago D. (2015). Social cognitive predictors of first and non-first-generation college students’ academic and life satisfaction. J. Couns. Psychol..

[18] Sheu H.B., Mejia A., Rigali-Oiler M., Primé D.R., Chong S.S. (2016). Social cognitive predictors of academic and life satisfaction: Measurement and structural equivalence across three racial/ethnic groups. J. Couns. Psychol..

[19] Oyserman D., Coon H.M., Kemmelmeier M. (2002). Rethinking individualism and collectivism: Evaluation of theoretical assumptions and meta-analyses. Psychol. Bull..

[20] Jiang J., Zeng T., Zhang C., Wang R. (2018). The mediating role of relatedness need satisfaction in the relationship between charitable behavior and well-being: Empirical evidence from China. Int. J. Psychol..

[21] Zhuang G., Xi Y., Tsang A.S.L. (2010). Power, conflict, and cooperation: The impact of guanxi in Chinese marketing channels. Ind. Mark. Manag..

[22] Heine S.J., Proulx T., Vohs K.D. (2006). The Meaning Maintenance Model: On the Coherence of Social Motivations. Personal. Soc. Psychol. Rev..

[23] Steger M.F., Oishi S., Kashdan T.B. (2009). Meaning in life across the life span: Levels and correlates of meaning in life from emerging adulthood to older adulthood. J. Posit. Psychol..

[24] Hillson J.M.C. An Investigation of Positive Individualism and Positive Relations with Others: Dimensions of Positive Personality. (Order No. AAMNQ28490). APA PsycInfo®. (619438953; 1999-95002-010). 1999. Ph.D. Thesis. https://www.proquest.com/dissertations-theses/investigation-positive-individualism-relations/docview/619438953/se-2?accountid=8554.

[25] Douglas K. (2012). Homo virtuous: The evolution of good and evil. New Sci..

[26] Neumann C.S., Kaufman S.B., Brinke L., Yaden D.B., Hyde E., Tsykayama E. (2020). Light and dark trait subtypes of human personality—A multi-study person-centered approach. Personal. Individ. Differ..

[27] Kaufman S.B., Yaden D.B., Hyde E., Tsukayama E. (2019). The Light vs. Dark Triad of Personality: Contrasting Two Very Different Profiles of Human Nature. Front. Psychol.

[28] Connor-Smith J.K., Flachsbart C. (2007). Relations between personality and coping: A meta-analysis. J. Personal. Soc. Psychol..

[29] Karademas E.C. (2006). Self-efficacy, social support and well-being The mediating role of optimism. Personal. Individ. Differ..

[30] Karademas E.C. (2007). Positive and negative aspects of well-being: Common and specific predictors. Personal. Individ. Differ..

[31] Ficková E. (2009). Reactive and proactive coping with stress in relation to personality dimensions in adolescents. Stud. Psychol..

[32] Roesch S.C., Wee C., Vaughn A.A. (2006). Relations between the Big Five personality traits and dispositional coping in Korean Americans: Acculturation as a moderating factor. Int. J. Psychol..

[33] Van Groningen A.J., Grawitch M.J., Lavigne K.N., Palmer S.N. (2021). Every cloud has a silver lining: Narcissism’s buffering impact on the relationship between the dark triad and well-being. Personal. Individ. Differ..

[34] Aghababaei N., Błachnio A. (2015). Well-being and the Dark Triad. Personal. Individ. Differ..

[35] Helzer E.G., Furr R.M., Hawkins A., Barranti M., Blackie L.E., Fleeson W. (2014). Agreement on the perception of moral character. Personal. Soc. Psychol. Bull..

[36] Goodwin G.P., Piazza J., Rozin P. (2014). Moral character predominates in person perception and evaluation. J. Personal. Soc. Psychol..

[37] Jiao L., Yang Y., Guo Z., Xu Y., Zhang H., Jiang J. (2021). Development and validation of the good and evil character traits (GECT) scale. Scand. J. Psychol..

[38] Jiao L., Yang Y., Xu Y., Gao S., Zhang H. (2019). Good and evil in Chinese culture: Personality structure and connotation. Acta Psychol. Sin..

[39] Haidt J., Graham J.J.S.J.R. (2007). When Morality Opposes Justice: Conservatives Have Moral Intuitions that Liberals may not Recognize. Soc. Justice Res..

[40] Lerner R.M., Lerner J.V., Lewin-Bizan S., Bowers E.P., Boyd M.J., Mueller M.K. (2011). Positive youth development: Processes, programs, and problematics. J. Youth Dev..

[41] Benson P.L., Scales P.C., Syvertsen A.K. (2011). The contribution of the developmental assets framework to positive youth development theory and practice. Adv. Child Dev. Behav..

[42] Zhou Z., Shek Daniel T.L., Zhu X.L. (2021). The Influence of Moral Character Attributes on Adolescent Life Satisfaction: The Mediating Role of Responsible Behavior. Child Indic. Res..

[43] Xie X., Wang Y., Gu S., Wei L.J., Ai P.S. (2017). Is altruism just other-benefiting? A dual pathway model from an evolutionary perspective. Adv. Psychol. Sci..

[44] Hu T.Y., Li J., Jia H., Xie X. (2016). Helping Others, Warming Yourself: Altruistic Behaviors Increase Warmth Feelings of the Ambient Environment. Front. Psychol..

[45] Sarason B.R., Sarason I.G., Pierce G.R. (1990). Traditional Views of Social Support and Their Impact on Assessment. Social Support: An Interactional View.

[46] Kong F., Yang K., Yan W., Li X.J. (2021). How Does Trait Gratitude Relate to Subjective Well-Being in Chinese Adolescents? The Mediating Role of Resilience and Social Support. J. Happiness Stud..

[47] Pocnet C., Antonietti J.P., Strippoli M.F., Glaus J., Preisig M., Rossier J. (2016). Individuals’ quality of life linked to major life events, perceived social support, and personality traits. Qual. Life Res..

[48] Helzer E.G., Critcher C.R. (2018). What Do We Evaluate when We Evaluate Moral Character.

[49] Hill P.L., Payne B.R., Jackson J.J., Stine-Morrow E.A., Roberts B.W. (2014). Perceived social support predicts increased conscientiousness during older adulthood. J. Gerontol. Ser. B Psychol. Sci. Soc. Sci..

[50] Huang J., Wang X., Li W., An Y. (2019). The relationship between conscientiousness and posttraumatic stress disorder among young Chinese firefighters: The mediating effect of perceived social support. Psychiatry Res..

[51] Ryan R.M., Deci E.L. (2001). On happiness and human potentials: A review of research on hedonic and eudaimonic well-being. Annu Rev. Psychol..

[52] Antonucci T.C., Birditt K.S., Akiyama H., Bengston V.L., Gans D., Pulney N.M., Silverstein M. (2009). Convoys of Social Relations: An Interdisciplinary Approach. Handbook of Theories of Aging.

[53] Cohen S., Wills T.A. (1985). Stress, social support, and the buffering hypothesis. Psychol. Bull..

[54] Zhu H. (2015). Social support and affect balance mediate the association between forgiveness and life satisfaction. Soc. Indic. Res..

[55] Kong F., Zhao J., You X. (2012). Social support mediates the impact of emotional intelligence on mental distress and life satisfaction in chinese young adults. Personal. Individ. Differ..

[56] Steger M.F., Kashdan T.B., Sullivan B.A., Lorentz D. (2008). Understanding the search for meaning in life: Personality, cognitive style, and the dynamic between seeking and experiencing meaning. J. Personal..

[57] Demirbaş-Çelik N., Keklik İ. (2019). Personality factors and meaning in life: The mediating role of competence, relatedness and autonomy. J. Happiness Stud.: Interdiscip. Forum Subj. Well-Being.

[58] Van Tongeren D.R., Green J.D., Hook J.N., Davis D.E., Davis J.L., Ramos M. (2015). Forgiveness increases meaning in life. Soc. Psychol. Personal. Sci..

[59] Lightsey O.R., Boyraz G., Ervin A., Rarey E.B., Gharibian G.G., Maxwell D. (2014). Generalized self-efficacy, positive cognitions, and negative cognitions as mediators of the relationship between conscientiousness and meaning in life. Can. J. Behav. Sci. /Rev. Can. Des Sci. Du Comport..

[60] Tiliouine H., Belgoumidi A. (2009). An exploratory study of religiosity, meaning in life and subjective wellbeing in muslim students from algeria. Appl. Res. Qual. Life.

[61] Boyraz G., Lightsey O.R., Can A. (2013). The Turkish version of the Meaning In Life Questionnaire: Assessing the measurement invariance across Turkish and American adult samples. J. Personal. Assess..

[62] Steger M.F., Frazier P., Oishi S., Kaler M. (2006). The meaning in life questionnaire: Assessing the presence of and search for meaning in life. J. Couns. Psychol..

[63] Glaw X., Kable A., Hazelton M., Inder K. (2017). Meaning in Life and Meaning of Life in Mental Health Care: An Integrative Literature Review. Issues Ment. Health Nurs..

[64] Li J., Dou K., Liang Y. (2021). The relationship between presence of meaning, search for meaning, and subjective well-being: A three-level meta-analysis based on the meaning in life questionnaire. J. Happiness Stud.: Interdiscip. Forum Subj. Well-Being.

[65] Jia N., Li W., Zhang L., Kong F. (2021). Beneficial effects of hedonic and eudaimonic motivations on subjective well-being in adolescents: A two-wave cross-lagged analysis. J. Posit. Psychol..

[66] Qiu L., Zheng X., Wang Y. (2008). Revision of the Positive and Negative Affect Scale. Chin. J. Appl. Psychol..

[67] Zimet G.D., Powell S.S., Farley G.K., Werkman S., Berkoff K. (1990). Psychometric characteristics of the multidimensional scale of perceived social support. J. Personal. Assess..

[68] Maiti T. (2006). Principles and practice of structural equation modeling. J. Am. Stat. Assoc..

[69] Zhao J., Gao F., Xu Y., Sun Y., Han L. (2020). The relationship between shyness and aggression: The multiple mediation of peer victimization and security and the moderation of parent–child attachment. Personal. Individ. Differ..

[70] Hayes A.F. (2013). Introduction to Mediation, Moderation, and Conditional Process Analysis: A Regression-Based Approach.

[71] Lin Y., Xiao H., Lan X., Wen S., Bao S. (2020). Living arrangements and life satisfaction: Mediation by social support and meaning in life. BMC Geriatr..

[72] Moreira P.A., Cloninger C.R., Dinis L. (2015). Personality and well-being in adolescents. Front Psychol..

[73] Klein N. (2017). Prosocial behavior increases perceptions of meaning in life. J. Posit. Psychol..

